# Comparison between a phenomenological approach and a morphoelasticity approach regarding the displacement of extracellular matrix

**DOI:** 10.1007/s10237-022-01568-3

**Published:** 2022-04-10

**Authors:** Q. Peng, W. S. Gorter, F. J. Vermolen

**Affiliations:** 1 Mathematical Institute, Leiden University, 2333 CA Niels Bohrweg, The Netherlands; 2 Delft Institute of Applied Mathematics, Delft University of Technology, Mekelweg 4, 2628 CD Delft, The Netherlands; 3 Computational Mathematics Group, Discipline group Mathematics and statistics, Faculty of Science, Hasselt University, Campus Diepenbeek, Agoralaan Gebouw D, BE 3590 Diepenbeek, Belgium

**Keywords:** Wound healing, Skin contractions, Phenomenological approach, Morphoelasticity, Force balance, Mechanics

## Abstract

Plastic (permanent) deformations were earlier, modeled by a phenomenological model in Peng and Vermolen (Biomech Model Mechanobiol 19(6):2525–2551, 2020). In this manusctipt, we consider a more physics-based formulation that is based on morphoelasticity. We firstly introduce the morphoelasticity approach and investigate the impact of various input variables on the output parameters by sensitivity analysis. A comparison of both model formulations shows that both models give similar computational results. Furthermore, we carry out Monte Carlo simulations of the skin contraction model containing the morphoelasticity approach. Most statistical correlations from the two models are similar, however, the impact of the collagen density on the severeness of contraction is larger for the morphoelasticity model than for the phenomenological model.

**Supplementary Information:**

The online version contains supplementary material available at 10.1007/s10237-022-01568-3.

## Introduction

Generally speaking, wound healing contains four overlapping phases for the skin to cure itself after getting injured. It is a complicated process with many biological elements involved, for instance, cells, cytokines and tissue molecules. They collaborate in the form of biological events, which contribute to the resurfacing, reconstitution and restoration of the tensile strength of the injured skin. Superficial skin injuries, where only the epidermis is damaged, heal without any problem. However, deeper wounds, where the damage extends into the dermal layer, cause more complications. The severeness depends on the size and depth of the wound.

In many cases, deep tissue injury causes contraction of the damaged skin tissue. Severe contractions that cause loss of mobility of joints are commonly referred to as contractures. Hence, contractures usually occur with disabilities of the joints of the patient. Contractures take place due to the large traction forces exerted by the (myo)fibroblasts on the extracellular matrix. Skin contraction occurs in the proliferation phase, which continues for two to four weeks post wounding. The contraction process is characterized by active proliferation and differentiation of fibroblasts. The main healing mechanisms in this phase are re-epithelialization, fibroplasia, angiogenesis and the development of granulation tissue. During this stage, fibroblasts are attracted to the damaged region by a number of chemokines like platelet-derived growth factor (PDGF) and transforming growth factor-beta (TGF-beta). Induced by the high concentration of TGF-beta, some fibroblasts differentiate into myofibroblasts, which is a cell phenotype having the properties of both fibroblasts and smooth muscle cells (Darby et al. 2014; Grinnell 1994; Phan 2008; Chaudhari 2015). Both fibroblasts and myofibroblasts are responsible cell phenotypes for the regeneration of collagen bundles and the reestablishment of the extracellular matrix (ECM). Compared to fibroblasts, the myofibroblasts exert even larger forces on the ECM and secret more collagen bundles, and as a result of myofibroblasts, the damaged skin contracts more. For acute wounds, myofibroblasts are often found to persist, which are otherwise supposed to be extinct as a result of apoptosis (programmed death) (Frangogiannis 2017; Xue and Jackson 2015). The amount of contraction is related to the size, shape, depth and anatomical location of the wound. For instance, tissues with stronger laxity contract more than loose tissues, furthermore square-shaped wounds contract more than circular ones (Enoch and Leaper 2008; Li and Wang 2011). Usually, a reduction by $5-10\%$ of the original wound volume is observed in clinical studies. For a more detailed biological description on wound healing, we refer to Enoch and Leaper (2008); Li and Wang (2011).

Many different models have been developed for all the phases of wound healing, see for instance Boon et al. (2016); Koppenol (2017); Cumming et al. (2009a); Murray (2003); Ziraldo et al. (2013); Cohen and Mast (1990). These models can be categorized as agent-based models and continuum-based models. Since we are interested in the cause of contractions and contractures, an agent-based model is preferred, since all the cellular events and cell positions can be tracked explicitly. To model the traction forces exerted by the (myo)fibroblasts, we divide the cell boundary into line segments, then use a point force at the midpoint of every boundary segment. Regarding the deformation of the ECM, there are two different approaches, namely a phenomenological approach and a morphoelasticity approach.

In Peng and Vermolen (2020), we modelled the traction forces exerted by the (myo)fibroblasts with a phenomenological approach, in which the virtual forces are applied at the line segments of the mesh elements. An alternative approach to model plastic deformation is by the use of morphoelasticity, which is a more advanced model. Morphoelasticity is widely used in biological modelling to describe elastic growth, for instance, the growth of tumors (Goriely and Moulton 2011), the growth of seashells (Rudraraju et al. 2019) and contractions of scars after an injury (Koppenol 2017; Ben Amar et al. 2015) etc. Morphoelasticity provides a description of the evolution of plastic (permanent) deformation.

This manuscript is structured as follows: we firstly introduce both approaches in Sect. 2. Section 4 describes the investigation of the impact of various parameters on the morphoelasticity approach. Subsequently, we show some numerical results in two dimensions to compare these two approaches in Sect. 5. In Sect. 6, we perform a Monte Carlo based parameter sensitivity analysis on the basis of the morphoelastic approach for the displacement of the extracellular matrix (ECM). Finally, Sect. 7 presents the conclusions.

## Mathematical models

In this section, mainly the force balance part of the skin contraction model will be discussed. For the sake of completeness of this manuscript, we present a brief summary of the skin contraction model developed in Peng and Vermolen (2020), which contains all the other mechanics like chemotaxis and interaction between cells etc.

**Fig. 1:**
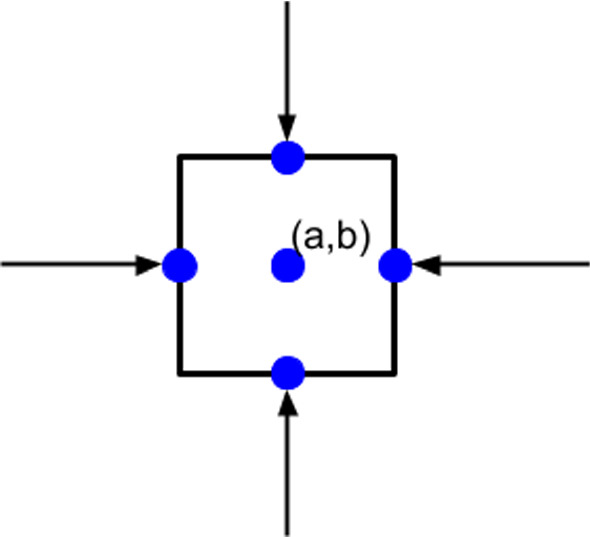
A schematic of the cellular traction forces exerted by a cell, which is approximated by a square

To model the force exerted by a (myo)fibroblast, we divide the cell boundary into line segments, then a point force is applied at the midpoint of each line segment, which points inward to the center of the cell; see Figure 1 where we use a square to approximate the cell and the point forces are applied on each line segments. The point force is decribed by the Dirac delta distribution, which is defined in any dimensionality by

$$\begin{aligned} \delta ({\varvec{x}}) = 0, \text{ for } {\varvec{x}}\ne {\varvec{0}}, \end{aligned}$$

and constrained to satisfy the identity that

$$\begin{aligned} \int _{\Omega }\delta ({\varvec{x}})d\Omega =1, \text{ if } {\varvec{0}}\in \Omega \subset {\mathbb {R}}^n. \end{aligned}$$

Here, $\Omega$ is an open subset of ${\mathbb {R}}^n$. Therefore, we use the expression in Peng and Vermolen (2020) to describe the total forces exerted actively by all the cells in the computational domain:

1 $$\begin{aligned} {\varvec{f}}_t({\varvec{x}};t)=\sum _{i=1}^{T_N(t)}\sum _{j=1}^{N_S^i}P({\varvec{x}}_j^i,t){\varvec{n}}({\varvec{x}}_j^i(t))\delta ({\varvec{x}}-{\varvec{x}}_j^i(t))\Delta \Gamma _N^{i,j}, \end{aligned}$$

where $T_N(t)$ is the number of cells at time *t*, $N_S^i$ is the number of line segments of cell *i*, $P({\varvec{x}},t)$ is the magnitude of the pulling force exerted at point ${\varvec{x}}$ and time *t* per length, ${\varvec{n}}({\varvec{x}})$ is the unit inward pointing normal vector (towards the cell centre) at position ${\varvec{x}}$, ${\varvec{x}}_j^i(t)$ is the midpoint on line segment *j* of cell *i* at time *t* and $\Delta \Gamma _N^{i,j}$ is the length of line segment *j*. Note that the above equation represents the cellular forces in case of a general polygonal (in ${\mathbb {R}}^2$) or a general polyhedral (in ${\mathbb {R}}^3$) division of the cell boundary. This method is also used in fluid dynamics and is known as the immersed boundary method. Theoretically, as $N_S^i\rightarrow \infty$, i.e. $\Delta \Gamma _N^{i,j}\rightarrow 0$, Eq (1) becomes (Boon et al. 2016)

2 $$\begin{aligned} {\varvec{f}}_t({\varvec{x}};t)=\sum _{i=1}^{T_N(t)}\int _{\partial \Omega _N^i}P({\varvec{x}}^i,t){\varvec{n}}({\varvec{x}}^i(t))\delta ({\varvec{x}}-{\varvec{x}}^i(t))d\Gamma _N^{i}, \end{aligned}$$

Here, ${\varvec{x}}^i(t)$ is a point on the cell boundary of cell *i* at time *t*. In the current manuscript, we do not consider any shape changes of cells, and therefore, we divide the boundary of each cell in four segments on which a force towards the center is exerted. This has been visualized in Figure 1. This approach has been used in both the earlier modelling framework in Peng and Vermolen (2020) and the current modelling framework.

### Phenomenological approach

In Peng and Vermolen (2020), a phenomenological model was developed to simulate permanent deformations of skin. The idea was based on the inclusion of artificial forces on the faces (that is, boundary segments) of the mesh elements. This approach is motivated by the fact that it is known that myofibroblasts shorten the polymeric chains that constitute the extracellular matrix. The phenomenological forces on the boundary of mesh elements are also referred to as ’virtual forces’ or ’plastic forces’. The latter qualification is motivated by the permanent nature of these forces. In Peng and Vermolen (2020); Koppenol (2017), the magnitude of the virtual forces in a mesh element is determined by the duration that myofibroblasts occupy the region of the mesh element. The principle behind the determination is based on solving a ordinary differential equation that leads to a chemical equilibrium that reflects the shortening of the polymeric chains. In this manuscript, our focus lies on the qualitative comparison between two approaches, hence, we simplify the phenomenological approach here by assuming the magnitude of the virtual force to be constant and to be equal to *Q*. For the plastic forces in the phenomenological model, we set

3 $$\begin{aligned} {\varvec{f}}_p({\varvec{x}};t)=\sum _{i=1}^{N_E}\sum _{e=1}^{N_e^i}Q{\varvec{n}}({\varvec{x}})\delta ({\varvec{x}}-{\varvec{x}}_e^i(t))\Delta \Gamma _E^{i,e}, \end{aligned}$$

where $N_E$ is the total number of mesh triangular elements, $N_e^i$ is the total number of edges of mesh elements (hence for triangular elements, $N_e^i = 3$), *Q* is the magnitude of the plastic force density of myofibroblast, ${\varvec{n}}({\varvec{x}})$ is the unit inward (hence directed towards the center of the mesh element) pointing normal vector, ${\varvec{x}}_e^i(t)$ is the midpoint of the boundary segment in consideration and $\Delta \Gamma _E^{i,e}$ is the length of the boundary segment, respectively. The virtual force (${\varvec{f}}_p({\varvec{x}}; t)$) has the similar form as the expression of the total force exerted by the cell (see Eq (1)), however, the virtual force is applied on the mesh line segments to describe the permanent force, which simulates the residual forces even after the cell dies and has disappeared from the computational domain. Therefore, this virtual force only occurs in the phenomenological model but not in the morphoelastic model.

We note that this virtual force framework is sufficiently general to extend to different element shapes. Furthermore, we assume that the displacement of the tissue vanishes far away and hence a homogeneous Dirichlet boundary condition is used. Hence, the boundary value problem is expressed by

4 $$\begin{aligned} \left\{ \begin{aligned}&-\nabla \cdot \varvec{\sigma }={\varvec{f}}_t+{\varvec{f}}_p, \text{ in } \Omega \text{, }\\&{\varvec{u}}({\varvec{x}},t)={\varvec{0}}, \text{ on } \partial \Omega \text{. } \end{aligned} \right. \end{aligned}$$

Here, $\varvec{\sigma }$ contains both elastic and viscoelastic part, that is,

5 $$\begin{aligned} \begin{aligned} \varvec{\sigma }&= \varvec{\sigma }_{elas}+\eta \varvec{\sigma }_{visco}\\&= \frac{E_s}{1+\nu }\left\{ \varvec{\epsilon }+tr(\varvec{\epsilon })\left[ \frac{\nu }{1-2\nu }\right] {\varvec{I}}\right\} \\&+ \eta [\mu _1\dot{\varvec{\epsilon }}+\mu _2\nabla \cdot \dot{{\varvec{u}}}{\varvec{I}}]. \end{aligned} \end{aligned}$$

Furthermore, $\varvec{\epsilon }$ is the infinitesimal strain tensor as defined by

6 $$\begin{aligned} \varvec{\epsilon } = \frac{1}{2}(\nabla {\varvec{u}}+(\nabla {\varvec{u}})^T), \end{aligned}$$

and ${{\,\mathrm{tr}\,}}(\varvec{\epsilon })$ is the trace of the tensor, and ${\varvec{f}}_t$ and ${\varvec{f}}_p$ are defined by Eq (1) and Eq (3), respectively. Furthermore, $\dot{\varvec{\epsilon }}$ and $\dot{{\varvec{u}}}$ represent the time derivative of $\varvec{\epsilon }$ and ${\varvec{u}}$, respectively.

To model a wound, a subdomain $\Omega _w \subset \Omega$ (strict subset) is chosen. Further, zero initial displacement is assumed, that is ${\varvec{u}}({\varvec{x}},t) = {\varvec{0}}$.

### Morphoelasticity approach

Whereas the phenomenological approach is based on directly solving the equations for the local displacement of the tissue, the morphoelastic approach represents partial differential equations for the displacement velocity and the effective strain $\varvec{\epsilon }$. Note that the effective strain does not necessarily satisfy Eq (6). To this extent, the equations for the balance of momentum and the evolution of the strain tensor are given by:

7 $$\begin{aligned} \left\{ \begin{aligned}&\rho \left[ \frac{D{\varvec{v}}}{Dt}+{\varvec{v}}(\nabla \cdot {\varvec{v}})\right] -\nabla \cdot \varvec{\sigma }={\varvec{f}}_t, \text{ in } \Omega \text{, }\\&\frac{D\varvec{\epsilon }}{Dt}+\varvec{\epsilon }{{\,\mathrm{skw}\,}}({\varvec{L}})-{{\,\mathrm{skw}\,}}({\varvec{L}})\varvec{\epsilon }+[{{\,\mathrm{tr}\,}}(\varvec{\epsilon })-1]{{\,\mathrm{sym}\,}}({\varvec{L}})=-\alpha \varvec{\epsilon },\\&\text{ in } \Omega \text{, }\\&{\varvec{v}}({\varvec{x}},t)={\varvec{0}}, \text{ on } \partial \Omega \text{, } \end{aligned} \right. \end{aligned}$$

where $\rho$ is the density of the extracellular matrix, ${\varvec{f}}_t$ is the temporary force described in Eq (1), ${\varvec{L}}=\nabla {\varvec{v}}$ and $\alpha$ is a non-negative constant. Here, $\displaystyle \frac{D{\varvec{y}}}{Dt} = \frac{\partial {\varvec{y}}}{\partial t}+{\varvec{v}}\nabla {\varvec{y}}$ is the material derivative for any tensor field ${\varvec{y}}$ and ${\varvec{v}}$ is the displacement velocity. Furthermore, ${{\,\mathrm{skw}\,}}({\varvec{L}})=\displaystyle \frac{1}{2}({\varvec{L}}-{\varvec{L}}^T)$ is the skew-symmetric and ${{\,\mathrm{sym}\,}}({\varvec{L}})=\displaystyle \frac{1}{2}({\varvec{L}}+{\varvec{L}}^T)$ is the symmetric part of the tensor, respectively, and ${{\,\mathrm{tr}\,}}(\varvec{\epsilon })$ represents the trace of $\varvec{\epsilon }$. The definition of the stress tensor in the morphoelasticity approach has the same components as in the phenomenological approach in Eq (5). Since one solves for the displacement velocity and the effective strain in the morphoelastic approach, the viscous part in Eq (5) is replaced with

8 $$\begin{aligned} \varvec{\sigma }_{visco} = \mu _1{{\,\mathrm{sym}\,}}({\varvec{L}})+\mu _2{{\,\mathrm{tr}\,}}({{\,\mathrm{sym}\,}}({\varvec{L}})){\varvec{I}}. \end{aligned}$$

The initial boundary value problem for morphoelasticity is nonlinear and requires the solution of the displacement velocity *v* and the strain tensor $\varvec{\epsilon }$. This is in contrast to the boundary value problem in Eq (4). Since we are interested in the contraction of the scar region $\Omega _w$, we approximate the displacement by integrating the velocity over time: ${\varvec{u}}(t)=\int _{0}^{t}{\varvec{v}}(s)ds$.

## A summary of the skin contraction model

For the sake of the completeness of this paper, we briefly present the model developed in Peng and Vermolen (2020). The model contains various biological segments, for instance, signalling molecules, cells and tissue bundles. For a more detailed description of the skin contraction model, we refer to our previous work (Peng and Vermolen 2020).

### Signalling molecules

There are three categories of signalling molecules in the model. The concentration of each signalling molecules is expressed by the convection-diffusion equation:

$$\begin{aligned} \frac{\partial c}{\partial t}+\nabla \cdot [c\cdot {\varvec{v}}(t,{\varvec{x}}(t))]-D\Delta c=F, \end{aligned}$$

with Robin’s boundary condition:

$$\begin{aligned} \frac{\partial c}{\partial {\varvec{n}}}+\kappa _c c=0, \end{aligned}$$

where *c* is the concentration of the signalling molecule, *D* is the diffusion rate, ${\varvec{v}}(t, {\varvec{x}}(t))$ is the displacement velocity of the ECM (solved from one of the force balance models in Section 2), *F* is the reaction term depending on the category of the signalling molecules, and $\kappa _c$ is a positive constant. To distinguish between the cytokines, we add the subscript “PDGF”, “TGF” or “tPA” if it is necessary (i.e. $c_{PDGF}$, $c_{TGF}$ and $c_{tPA}$). Speaking for the initial condition, PDGF has higher concentration in the wound region and lower in the undamaged region; there is no TGF-beta over the domain initially; the concentration of tPA is higher over the edge between the wound and undamaged skin and lower in the rest of the computational domain.

### Tissue bundles

Dallon et al. (1999) and Cumming et al. (2009b) developed a tensor-based representations of tissue bundles, such that the density and the orientation of the tissue bundles can be described. The tensor of the orientation of the tissue bundle is given by

$$\begin{aligned} \varvec{\Omega ^k}({\varvec{x}};t) = \int _{0}^{\pi }{\varvec{p}}(\theta ){\varvec{p}}(\theta )^T\rho ({\varvec{x}},t,\theta ), \end{aligned}$$

where *k* indicates the type of the tissue: $k = f$ for fibrin and $k = c$ for collagen, ${\varvec{p}}(\theta )^T = [\cos \theta ,\sin \theta ]$ is the unit vector in the direction $\theta$, and $\rho ({\varvec{x}},t,\theta )$ is the density of collagen at position ${\varvec{x}}$ and time *t*. Since the tensor is symmetric positive definite, it can be decomposed as the sum of weighed outer products of orthogonal eigenvectors, which in the two-dimensional case gives:

$$\begin{aligned} \varvec{\Omega }^k({\varvec{x}},t)&= \lambda _1({\varvec{x}},t){\varvec{v}}_1({\varvec{x}},t){\varvec{v}}_1({\varvec{x}},t)^T\\&+\lambda _2({\varvec{x}},t){\varvec{v}}_2({\varvec{x}},t){\varvec{v}}_2({\varvec{x}},t)^T, \end{aligned}$$

where $\lambda _1({\varvec{x}},t)$ and $\lambda _2({\varvec{x}},t)$ are eigenvalues, and ${\varvec{v}}_1({\varvec{x}},t)$ and ${\varvec{v}}_2({\varvec{x}},t)$ are corresponding eigenvectors.

We assume that the fibrin bundles are degradated by the concentration of tPA, hence, for every entry in $\varvec{\Omega }^f$, it is determined by

$$\begin{aligned} \frac{\partial \varvec{\Omega }_{ij}^f}{\partial t}+\nabla \cdot [\varvec{\Omega }_{ij}^f\cdot {\varvec{v}}(t, {\varvec{x}}(t))] = -\delta _\rho [c_{tPA}\varvec{\Omega }_{ij}^f], \end{aligned}$$

for any $i,j\in \{1,2\}$ and $\delta _\rho$ represents the degradation rate of fibrin bundles. The collagen bundles are deposited by the (myo)fibroblasts in the direction of active migration. Since the cells are much smaller than the computational domain, we use Dirac Delta functions to model the secretion of the collagen bundles. Furthermore, the secretion rate depends on the amount of total density including fibrin and collagen:

$$\begin{aligned} \left. \begin{aligned}&\frac{\partial \varvec{\Omega }^c_{ij}}{\partial t}+\nabla \cdot [\varvec{\Omega }^c_{ij}\cdot {\varvec{v}}(t,{\varvec{x}}(t))]=\sum _{k=1}^{T_N(t)}\left( 1-\alpha _\rho [\rho ^f({\varvec{x}}_N^k(t))\right. \\&\left. +\rho ^c({\varvec{x}}_N^k(t))]\right) [{\varvec{r}}_N^k(t)({\varvec{r}}_N^k(t))^T]_{ij}\delta ({\varvec{x}}-{\varvec{x}}_N^k(t)), \end{aligned} \right. \end{aligned}$$

where ${\varvec{r}}_N^k(t)=(d{\varvec{x}}^k_N(t)-{\varvec{v}}dt)/\Vert d{\varvec{x}}^k_N(t)-{\varvec{v}}dt\Vert$ is the unit vector of the direction of active displacement of (myo)fibroblast *k* at time *t* for any $i,j\in \{1,2\}$, and ${\varvec{x}}_N^k(t)$ is the centre position of (myo)fibroblast with index *k*. Initially, it is assumed that the collagen and fibrin are isotropic. Therefore, we have

$$\begin{aligned} \underline{\underline{\Omega }}^f_0=\frac{{\mathbb {I}}_{\Omega _w}}{2\alpha _\rho }\underline{\underline{I}}=\frac{{\mathbb {I}}_{\Omega _w}}{2\alpha _\rho } \begin{bmatrix} 1&{}0\\ 0&{}1 \end{bmatrix}, \end{aligned}$$

and

$$\begin{aligned} \underline{\underline{\Omega }}^c_0=\frac{1-{\mathbb {I}}_{\Omega _w}}{2\alpha _\rho }\underline{\underline{I}}=\frac{1-{\mathbb {I}}_{\Omega _w}}{2\alpha _\rho } \begin{bmatrix} 1&{}0\\ 0&{}1 \end{bmatrix}, \end{aligned}$$

where ${\mathbb {I}}_{\Omega _w}$ is the indicator function that was defined as

9 $$\begin{aligned} {\mathbb {I}}_{\Omega _w}= {\left\{ \begin{array}{ll} 1,\quad {\varvec{x}}\in \Omega _{w},\\ 0,\quad{\varvec{x}}\notin \Omega _{w}, \end{array}\right. } \end{aligned}$$

where $\Omega _{w}$ is the wound region as a subdomain in the computational domain. and $\alpha _\rho$ is a positive constant.

### Cellular activities and displacement of cells

In this model, we assume that only regular fibroblasts can proliferate and differentiate to myofibroblasts, while macrophages and myofibroblasts will only be subject to apoptosis (programmed death). These cellular events are modelled by stochastic process, which follow the exponential statistical distribution.

Cells migrate due to various mechanisms, for instance, chemotaxis, random walk, passive convection and interaction between cells. The collagen bundles also have an immediate impact on the migration of (myo)fibroblasts. For the immune cells, chemotaxis is associated with PDGF, then the displacement of the center position of *i*-th macrophage is given by

10 $$\begin{aligned} \left. \begin{aligned} d{\varvec{r}}_i(t)&=\alpha _i{\hat{M}}(\varvec{r_i})\varvec{{\hat{z}}_i}dt+\mu _{c}\frac{\nabla c_{PDGF}}{\Vert \nabla c_{PDGF}\Vert +\gamma }dt+{\varvec{v}}dt\\&\quad +\sigma _{rw} d{\varvec{W}}(t), \text{ for } \text{ all } i\in \left\{ 1,\dots ,n\right\} , \end{aligned} \right. \end{aligned}$$

where $\mu _{c}$ is the constant representing the weight of chemotaxis, which is expressed by

11 $$\begin{aligned} v\left( 1-\alpha _\rho \frac{\rho ^f+\rho ^c}{2}\right) , \end{aligned}$$

here, *v* is the speed of biased movement of cells, $c_{PDGF}$ is the concentration of PDGF which is initially high in injured region and low in uninjured region, $\gamma$ is a small positive constant to prevent the denominator from being zero and ${\varvec{v}}$ is the displacement velocity of the substrate, which follows from solving the momentum balance displacement.

For the (myo)fibroblasts, similar to the displacement of microphages, the new positionof the *i*-th (myo)fibroblast is updated via

$$\begin{aligned} {\varvec{r}}_i(t + \Delta t) = {\varvec{r}}_i(t) + d{\varvec{r}}_i(t), \end{aligned}$$

where $d{\varvec{r}}_i(t)$ is calculated from

12 $$\begin{aligned} \left. \begin{aligned} d{\varvec{r}}_i(t)&=\alpha _i{\hat{M}}(\varvec{r_i})\varvec{{\hat{z}}_i}dt+\mu _{c}\left\{ \left[ 1-\alpha _\rho \rho ^c({\varvec{r}}_i(t))\right] \underline{\underline{I}}\right. \\&\quad+\left. \left[ \alpha _\rho \rho ^c({\varvec{r}}_i(t))\underline{\underline{\Omega }}^c({\varvec{r}}_i(t))\right] \right\} \frac{\nabla c_{TGF}}{\Vert \nabla c_{TGF}\Vert +\gamma }dt\\&\quad+{\varvec{v}}dt+\sigma _{rw} d{\varvec{W}}(t), \text{ for } \text{ all } i\in \left\{ 1,\dots ,n\right\} , \end{aligned} \right. \end{aligned}$$

where $\mu _{c}$ is the same expression as before in Eq (11), and $c_{TGF}$ is the concentration of TGF-beta secreted by macrophages.

For a detailed description of the model and the explanation of all the parameters, we refer to our previous work Peng and Vermolen (2020).

### Comparison between two approaches

Both the phenomenological and the morphoelastic approaches can be used to simulate the permanent contraction of post-wounded skin. The two models have the agent-based character for the cell dynamics in common. The cellular dynamics regarding migration, division, differentiation and the proliferation of collagen, including its orientation are identical. The momentum balance differs between the two models in the sense that the phenomenological model explicitly computes the displacement at all positions in the computational domain. Furthermore, the permanent displacements are modelled by the use of ’apparent’, or ’virtual’ forces that arise depending on the exposure of an mesh element to myofibroblasts. The physics behind this assumption is motivated by the assumption that the myofibroblasts induce shortening of the polymeric chains, which leaves residual stresses around these fibers. The morphoelastic model is based on exposure to strain. A large exposure to strain, induces significant built-up of permanent deformation. The formalism that we used here is inspired from the model in Koppenol (2017), where morphoelasticity is considered in the context of post-trauma skin contraction. In that model, the strain and the number of Matrix metalloproteinases (MMPs) and myofibroblasts determine the extent of permanent contraction. The current modelling framework does not account for MMPs and hence uses a somewhat simplified relation between permanent displacement and local strain.

The similarities and differences between the morphoelastic model and the phenomenological model are summarized in Table 1. Firstly, the unknown parameters to be solved in these two models are different: one can obtain the displacement of the ECM directly by solving the linear system from the phenomenological model, however, one needs to approximate the displacement in the morphoelastic model, which is a nonlinear system solving both the velocity and the strain tensor of the domain. Secondly, the phenomenological model does not consider inertia, which the morphoelastic model does. Thirdly, the permanent displacements in the phenomenological model are solely determined by the history path of local presence of myofibroblasts (in a mesh element), and this is modelled in the balance of momentum directly, whereas the permanent displacements in the morphoelastic model follow from the history path of the local strain as a result of the forces exerted by both the myofibroblasts and the fibroblasts. In other words, the exposure to mechanical triggers invokes the permanent deformations in the morphoelastic formalism, whereas the actual presence of myofibroblasts triggers the permanent deformations in the phenomenological model. Speaking of the similarities, both models in this manuscript are agent-based models, therefore, they are mainly suitable for the microscale due to the high computational cost. Furthermore, both of them contain various stochastic processes regarding random walk, cell activities (i.e. proliferation, apoptosis and differentiation).

**Table 1:**
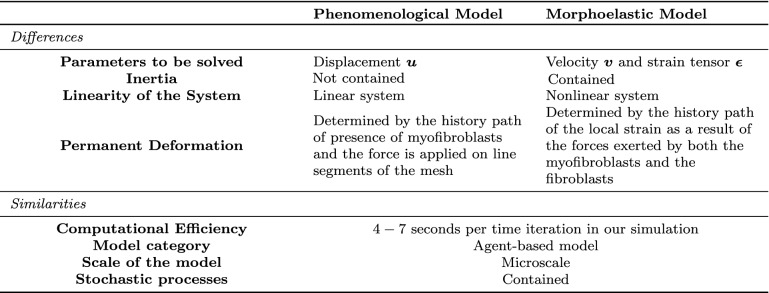
Similarities and differences between the phenomenological model and the morphoelastic model

## Sensitivity test of morphoelasticity approach

**Fig. 2:**
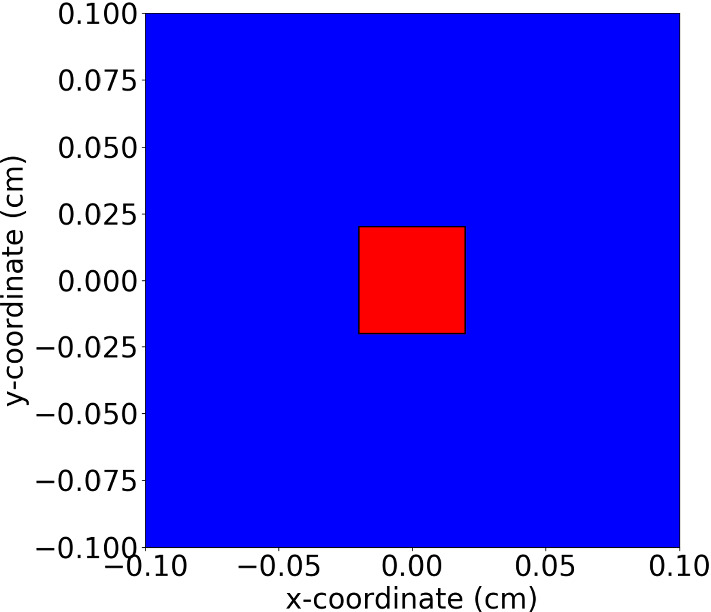
A schematic of the scar and healthy skin region is shown for the simulations in Sect. 4, where the computational domain is in the order of a tenth of centimeter. The blue region is the undamaged skin and the red region is the wound with dimensions of $(-0.02, 0.02)cm\times (-0.02, 0.02)cm$, which is strictly embedded in the computational domain in the size of $(-0.1, 0.1)cm\times (-0.1, 0.1)cm$

As the model mainly deals with the deformation of the wound, we define a subdomain (as scar region) in the centre of the computational domain (see Fig. 2) and we investigate the relative scar area at different times. Here, we define the relative scar (or wound) area by

$$\begin{aligned} r(t) = \frac{A_{\Omega _{w}}(t)}{A_{\Omega _{w}}(0)}, \end{aligned}$$

where $\Omega _w$ is the scar (or wound) region, $A(\Omega _w; t)$ is the area of $\Omega _w$ at time *t*. Since we are interested in the impact of the input parameters on the contraction of the skin tissue, we carry out sensitivity tests where we only change the value of one parameter. The parameter values are displayed in Table 2.

In this section, we assume that the shapes and sizes of the cells are identical, and that they are at fixed locations that are strictly within the wound region $\Omega _w\subset \Omega$. The traction forces are released at all times from $t= 5$ day. For reasons of comparison and presentation, we define $t_{min}$ as the time point at which the scar exhibits the maximal contraction, defined by $\max _{t\geqslant 0}(A_{\Omega _{w}}(0) - A_{\Omega _{w}}(t))$, that is, the area of the scar is minimal here:

13 $$\begin{aligned} t_{min}:=\arg \max _{t\geqslant 0}(A_{\Omega _w}(0) - A_{\Omega _w}(t)). \end{aligned}$$

Note that $t_{min} > 0$. We are further interested in how the scar flows back towards its (new) equilibrium. Since this adjusted equilibrium is an asymptotic value, we define $t_{end}$ by

14 $$\begin{aligned} \begin{aligned} t_{end}:=\arg \min _{t > t_{min}} \{&|A_{\Omega _w}(t+\Delta t) - A_{\Omega _w}(t)|\\&\leqslant 0.01 \Delta t |A_{\Omega _w}(t_{min}) - A_{\Omega _w}(0)| \}. \end{aligned} \end{aligned}$$

**Table 2:** Parameter values and the range of values of the parameters in the sensitivity test in Sect. 4

Parameter	Description	Value or range of the values		Unit
$x_0$	Length of the computational domain in x direction	0.2		cm
$y_0$	Length of the computational domain in y direction	0.2		cm
$x_w$	Length of the wound region in x direction	0.04		cm
$y_w$	Length of the wound region in y direction	0.04		cm
$\Delta t$	Time step	0.02		day
$\rho$	Density of the ECM	1.02		g/cm^3^
$\mu _1$	Shear viscosity	100		g/(cm day)
$\mu _2$	Bulk viscosity	100		g/(cm day)
*P*	Magnitude of the cellular traction force	200		g cm/day
$E_s$	Young’s modulus of the ECM	31	[20.0, 200.0]	g/(cm day^2^)
$\eta$	Weight of viscosity in viscoelasticity	1	[0.6, 3]	−
$\alpha$	Degree of the permanent deformation	0.2	[0.0, 1.0]	$\hbox{day}^{-1}$
$\nu$	Poisson ratio of the ECM	0.48	[0.118, 0.495]	−

In this manuscript, we use a finite-element method to solve all the boundary value problems. Regarding the time-integration, we use a backward Euler method. From the theory, it is known that smooth solutions would be subject to errors of the order ${\mathcal {O}}(h^2)$ in the $L^2$ norm of the numerical approximation and ${\mathcal {O}}(\Delta t)$.

The numerical solutions of most boundary value problems are naturally smooth and convergent, except for the force balance model. The numerical stability and convergence of morphoelasticity model has been discussed in our other work Peng, Vermolen and Weihs (2021): with the refinement of the mesh, the convergence rate of the $L^2-$norm of the solution to Eq (7) (i.e. the velocity) is computed, which is 1.899828112 that is close to the theoretical value 2.

**Fig. 3:**
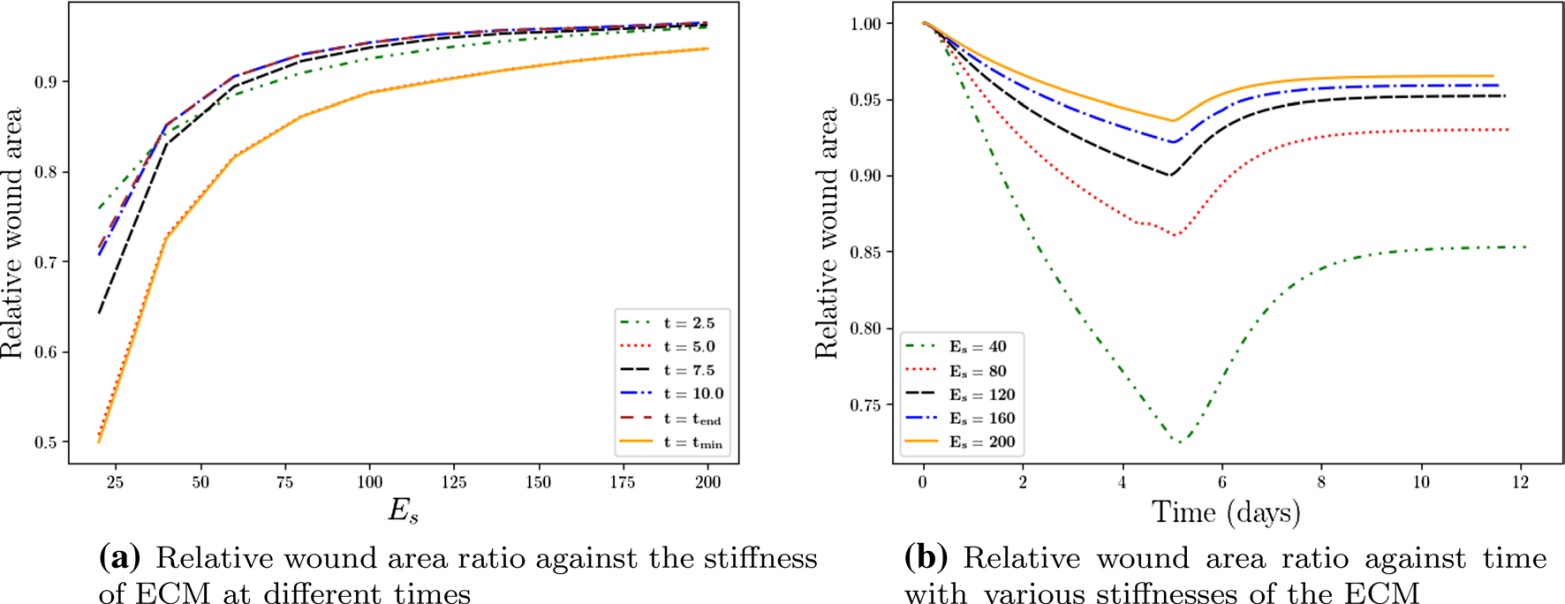
The impact of the stiffness $E_s$ is shown. Different curves stand for various times in subfigure (**a**) and various values of $E_s$ in subfigure (**b**), respectively

The Young’s modulus $E_s$ denotes the stiffness of the ECM. According to clinical observations (O’Leary et al. 2008), softer skins will develop a more serious skin contraction compared to stiffer skin. In other words, since the amount of contraction and displacement decreases with increasing stiffness ($E_s$), the scar area stays larger if the stiffness is larger. We show the results in Fig. 3 using various values of $E_s$. In Fig. 3a, the relative scar area is plotted as a function of $E_s$ and various colors of the curves stand for different time points. Figure 3b shows the relative scar area as a function of time and various colors of the curves stand for different values of $E_s$. It is concluded that the model confirms that a stiffer skin develops less contraction. Furthermore, Fig. 3a shows a hyperbolic correlation between the wound area and the stiffness.

**Fig. 4:**
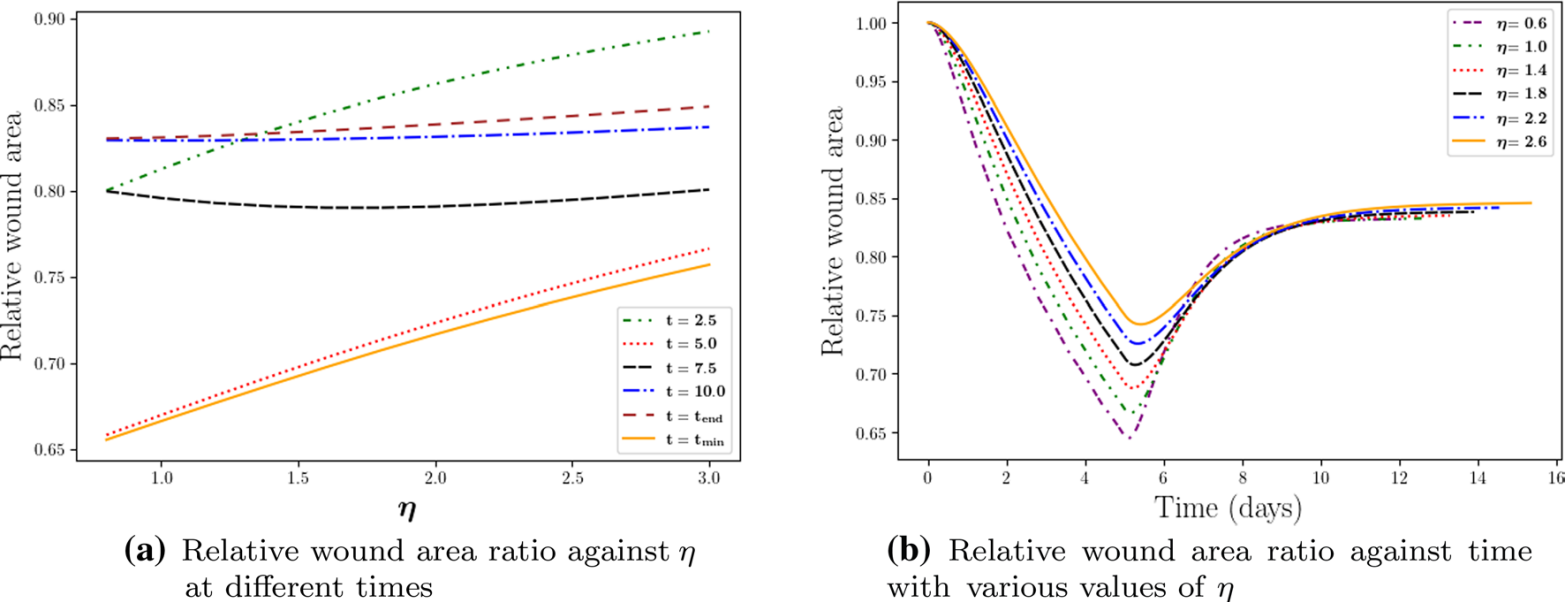
The impact of the weight of viscosity $\eta$ is shown. Different curves stand for various times in subfigure (**a**) and various values of $\eta$ in subfigure (**b**), respectively

**Fig. 5:**
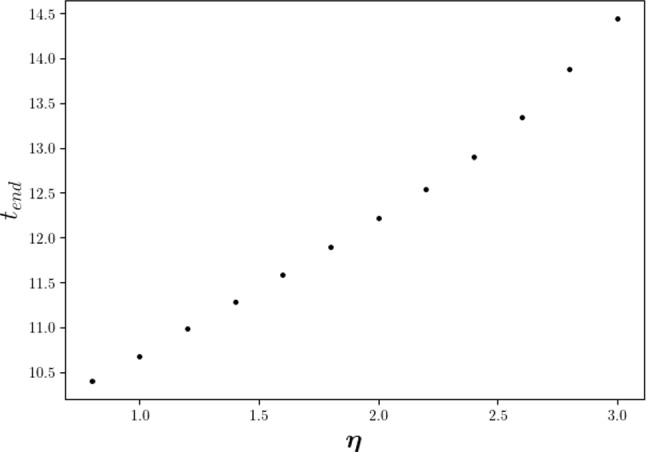
The impact of the weight $\eta$ of the viscous contribution to the stress tensor on the duration time of scar contraction, where $t_{end}$ is the moment defined in Eq (14) when the scar reaches its equilibrium

Skin is taken as a viscoelastic material (Kuwahara et al. 2008), that is, skin has both viscous and elastic properties. Viscosity is related to delayed recovery (or damping) from deformation, while elasticity is related to rebounding and quick recovery from deformation. Hence, we are interested in the impact of the damping property of the skin on the wound contraction. As it is shown in Fig. 4, with a larger viscosity $\eta$, the wound reaches its minimal area later and develops less contraction. In particular, there is an a weakly nonlinear correlation between the minimal wound area and the value of $\eta$. Similarly, for the recovering phase, a smaller viscosity results into faster recovery, that is, the model reaches the equilibrium earlier, i.e. at a smaller $t_{end}$. It can be seen from Fig. 5 that scar contraction takes place over a longer period with a linear tendency as $\eta$ increases. However, less significant differences appear in the final stages of scar contraction, and this tendency may be attributed to the fact that in the final stages relaxation takes place, where the equilibrium does not depend on the viscous part of the model.

**Fig. 6:**
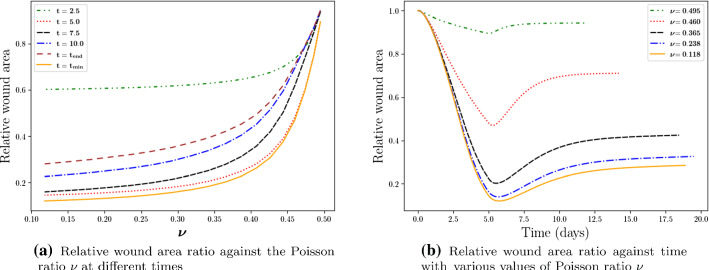
The impact of the Poisson ratio $\nu$ is shown. Different curves stand for various times in subfigure (**a**) and various values of $\nu$ in subfigure (**b**), respectively

The Poisson ratio $\nu$ indicates how much a material will deform in the direction perpendicular to the force direction. We vary $\nu$ here between (0.118, 0.495). Note that for incompressible materials, we have $\nu =0.5$. As the ECM becomes more incompressible, the wound area stays constant and this yields small contractions regardless of the time point post wounding. Furthermore, the curves in Fig. 6a show a hyperbolic behaviour. In general, a more compressible ECM indicates a higher degree of skin contraction and a larger healing time; see Fig. 6b.

**Fig. 7:**
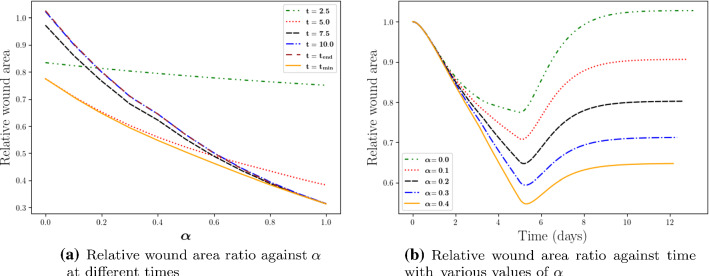
The impact of the degree of permanent deformation as a function of the morphoelasticity parameter $\alpha$ is shown. Different curves stand for various times in subfigure (**a**) and various values of $\alpha$ in subfigure (**b**), respectively

Different from the phenomenological approach, the degree of the permanent deformation in the morphoelasticity approach is determined by the value of $\alpha$ in Eq (7). Figure 7 displays simulation results with different values of $\alpha$. Generally speaking, a smaller value of $\alpha$ is beneficial to the scar since this leads to smaller final contractions. Note that when $\alpha = 0$, theoretically the wound will recover to its original size. However, due to numerical errors, in Fig. 7b, the final area ratio is slightly above 1. In fact the case $\alpha = 0$ reflects the viscoelastic case with the addition of inertia.

## Comparison between the phenomenological approach and the morphoelasticity approach

**Fig. 8:**
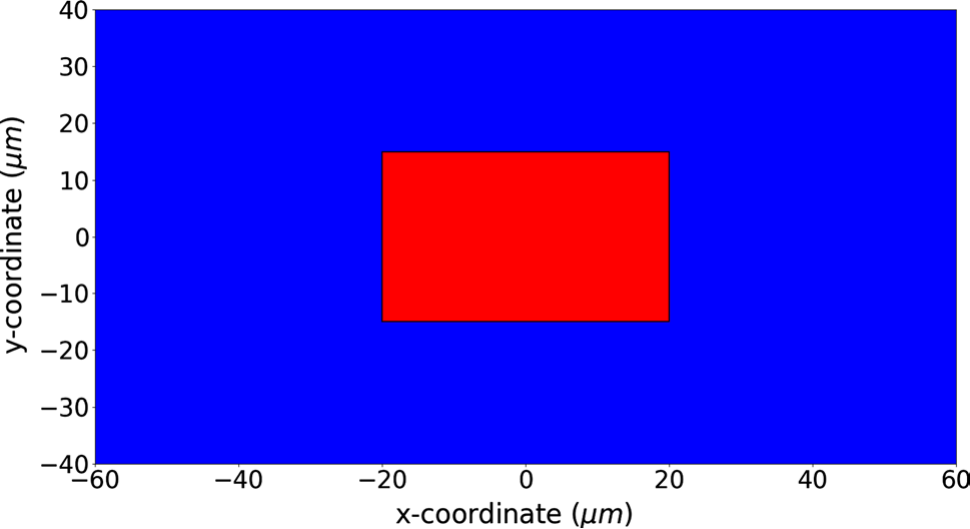
A schematic of the wound and healthy skin region for the simulations in Sect. 5, where the computational domain is in the order of a tenth of a micrometer. The blue region is the undamaged skin and the red region is the wound with dimensions $(-20, 20)\mu m\times (-15, 15)\mu m$, which is strictly embedded in the computational domain in the size of $(-60, 60)\mu m\times (-40, 40)\mu m$

**Fig. 9:**
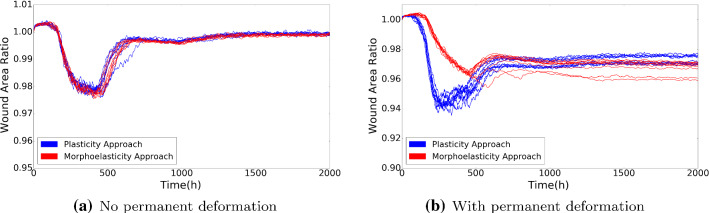
The plots show the wound area ratio with both phenomenological and morphoelasticity approach with and without permanent deformation, respectively. Red curves represent the morphoelasticity approach and blue curves represent the phenomenological approach. The parameter values are given in Tables 2 and 3

As both approaches are capable of reproducing the permanent plastic deformation during skin contraction, we are interested in whether they are numerically consistent, when both boundary value problems are solved by the finite-element methods with Lagrangian piecewise linear basis functions. We altered the skin contraction model in Peng and Vermolen (2020) by replacing the phenomenological model for plastic deformation (see Eq (4)) with the morphoelastic formulation (see Eq (7)). Most of the parameter values in the current study have been adopted from Peng and Vermolen (2020). These parameter values involve the properties of the cells and mechanical parameters of the ECM like the elasticity, viscosity and Poisson ratio. However, the current (new) model differs in the use of morphoelasticity. The parameters that were needed for this inclusion have not been taken from our earlier work. Hence, we only display the parameter values used in the force balance of the skin contraction model in Table 3. We have attempted to use realistic values for the input variables. However, some values of input variables are subject to large variations. The reasons for this variability are the patient-specific nature and poor availability of measurements that support these values. A typical example is the stiffness (Young’s modulus) of skin, which varies very much with the location in the body and varies a lot from patient to patient. Variations may even exceed a factor of 1000 (Liang et al. 2010; Trotta and Annaidh 2019; Wang and Lakes 2002). As a follow-up of our previous work in Peng and Vermolen (2020), we inherit the most settings and the initial domain is shown in Fig. 8. The results of the relative wound area against time are shown in Fig. 9.

**Table 3:** Parameter values of cells which will be used in the calculation of comparing two approaches in Section 5 of this manuscript

Parameter	Description	Value	Dimension	Reference
$E_s$	Substrate elasticity	100	$kg/(\mu m\;h^{2} )$	Liang et al. (2010)
$E_c$	Cell elasticity	5	$kg/(\mu m\;h^{2} )$	Dudaie et al. (2015)
*R*	Cell radius	2.5	$\mu m$	Dudaie et al. (2015)
$P_f$	Magnitude of temporary force of regular fibroblasts	2.08	$kg\;\mu m/h^{2}$	Koppenol (2017)
*Q*	Magnitude of plastic force of myofibroblasts in phenomenological approach	33	$kg\;\mu m/h^{2}$	Koppenol (2017)
$x_0$	Length of the computational domain in x direction	120	$\mu m$	Estimated in this study
$y_0$	Length of the computational domain in y direction	80	$\mu m$	Estimated in this study
$x_w$	Length of the wound region in x direction	40	$\mu m$	Estimated in this study
$y_w$	Length of the wound region in y direction	30	$\mu m$	Estimated in this study
$\Delta t$	Time step	0.1	*h*	Estimated in this study
$\nu$	Poisson’s ratio of the substrate	0.48	−	Estimated in this study
$\eta$	Weight of viscosity in viscoelasticity	10	$-$	Estimated in this study
$\mu _1$	Shear viscosity	16.89	$kg/(\mu m\;h)$	Estimated in this study
$\mu _2$	Bulk viscosity	11.26	$kg/(\mu m\;h)$	Estimated in this study
$P_m$	Magnitude of temporary force of myofibroblasts	10.4	$kg\;\mu m/h^{2}$	Estimated in this study
$\alpha$	Weight of the growth tensor in morphoelasticity approach if the permanent deformation exists	0.01	$h^{-1}$	Estimated in this study

We run several simulations with each approach since the model contains random processes. Figure 9 presents a case without and with permanent deformations (left and right subfigures, respectively). Figure 9a shows the case when plastic forces are not active in the phenomenological approach and the case that $\alpha = 0$ in the morphoelastic approach. In other words, after the forces being removed from the computational domain, the wound will recover to its original volume, that is, no contraction occurs if ${\varvec{f}}_p = {\varvec{0}}$ in the phenomenological approach and $\alpha =0$ in the morphoelastic approach. Figure 9b shows the results using the corresponding parameter values from Tables 2 and 3. In Fig. 9a, the curves of both colors are mostly overlapping, which indicates that both approaches can be used to describe scar contraction and as long as the main parameter values are the same, the models are more or less consistent. The inclusion of permanent deformations in both models by setting $\alpha$ and *Q* to positive values shows the same qualitative trends. However, it has turned out to be very difficult to make the curves from the two different models overlap, that is, make them agree quantitatively. We have carried out multiple attempts to reduce the quantitative difference between the results of both modelling frameworks. Either the minimal scar area from the two models agreed with other, or the final scar area did. We think that from a qualitative point of view the models predict the same kind of behavior. However, this difference shows that the two modelling frameworks are not the same. In case of the morphoelastic formulation, which was also used in the work by Koppenol (2017), the permanent deformation is solely modelled as a consequence of the strain (history path) that the tissue endures. The history path of the strain is caused by the forces that were exerted by both the fibroblasts and myofibroblasts. In case of the phenomenological approach, the permanent deformation is modelled by the actual presence of the myofibroblasts only, and not by the strain history path. This gives a different model formulation, though we observe similar evolution of skin. A next step would be to compare the outcomes from both modelling frameworks to small scale (*in vitro*) observations. Another difference between both modelling frameworks is in the balance of momentum. The balance of momentum in the morphoelastic approach involves inertia, which is more interesting from a numerical point of view, and also closer to physics, whereas inertia has not been accounted for in the phenomenological approach. The difference in the treatment of the balance of momentum does not give rise to significant differences between the modelling outcomes, see Fig. 9b. We finally remark that the differences between both modelling frameworks could possibly be reduced if the morphoelastic would only depend on the local presence of myofibroblasts. This was beyond the scope of our study.

Regarding computational efficiency, these two approaches take more or less the same CPU time for each iteration (around $4-7$s an Intel(R) Core(TM) i7-6500U CPU @ 2.50GHz computer). Hence in terms of computation time, there is no preference for either two models.

## Monte Carlo simulations with morphoelasticity

In the current simulations, we use the cell-based model as described in Peng and Vermolen (2020). Cells, treated as separate entities, are subject to migration (chemotaxis and random walk), proliferation, differentiation and apoptosis. Random walk is modeled by means of a vector Wiener process, and chemotaxis is modeled by an additional term migratory term that is proportional to the gradient of the TGF-beta. Furthermore, the random cellular processes such as apoptosis, cell differentiation and cell proliferation are modeled by the use of sampling from exponential distributions. More information can be found in Peng and Vermolen (2020). Therefore, the complete model contains a large amount of randomness due to the random processes like cell division and migration, and due to the uncertainty in the values of the large number of input parameters. The input parameters are descriptive for the cells, signalling molecules and tissue bundles. The outputs of interest include the scar area and collagen density at different times. We are, for instance, interested in the time at which the scar area reaches its (modified) equilibrium (i.e. when the scar area does not change significantly anymore; see Eq (14)), the time at which the contraction is maximal and the time at which the total amount of collagen reaches a certain threshold value. Note that, in this manuscript, the size of the cells and the scar have been set to academic values due to the limitation of computational resources. The values, as well as the statistical distributions of the input values are listed in Table 4. Other input values have been taken from Peng and Vermolen (2020).

**Table 4:** Distributions of the input parameters in Monte Carlo simulations when morphoelasticity approach is collaborated

Input Parameters				
Parameter	Description		Distributions	
$E\_s$	Substrate elasticity		Log normal$(\log 50, 0.1)$	
$\lambda _d$	Division rate of regular fibroblasts related to strain energy density		Uniform(1.5, 2.5)	
$\lambda _a$	Apoptosis rate of myofibroblasts		Normal(10, 0.1)	
$\lambda _{immune\_random}$	Rate of Point Poisson Process of macrophages random appearing on the edge between the wound and undamaged skin		Log normal$(\log 0.04,10^{-5})$	

Responses/Outputs				
Parameter	Description	Mean (Standard Deviation)	Monte Carlo Error	Relative Monte Carlo Error
*n*	The time when the model reaches equilibrium	657 (46.23974)	1.458587	0.00222
$n\_min$	The time when the wound has minimal volume	467.6 (39.80696)	1.255671	0.00269
$n\_rho\_0.5$	The first time when the ratio of average density of collagen exceeds 0.5	207.8 (4.77877)	0.150741	0.00073
$n\_rho\_0.8$	The first time when the ratio of average density of collagen exceeds 0.8	258.1 (5.292965)	0.641850	0.00249
$Area\_final$	The equilibrium wound area	1091.8 (46.70199)	1.473168	0.00135
$Area\_min$	The minimal wound area	1083.5 (47.81659)	1.508327	0.00139
$Area\_4d$	The wound area at 4th day after wounding	1199 (1.038872)	0.032770	0.00003
$Area\_rho\_0.5$	The wound area when the ratio of average density of collagen firstly exceeds 0.5	1171 (11.92360)	0.376118	0.00032
$Area\_rho\_0.8$	The wound area when the ratio of average density of collagen firstly exceeds 0.8	1149 (20.34776)	0.641850	0.00059
$rho\_c\_hat\_final$	The ratio of average density of collagen when the model reaches equilibrium	1.061 (0.0022738)	0.000173	0.00016
$rho\_c\_hat\_min$	The ratio of average density of collagen when the wound area is minimal	1.065 (0.0023577)	0.000744	0.00070
$rho\_c\_hat\_4d$	The ratio of average density of collagen at 4th day after wounding	0.05485 (0.00097955)	0.000309	0.00564

In total, 1005 samples are collected and the basic statistics, being the obtained output sample mean and output sample standard deviation, are shown in Table 4. With respect to the input variables, the stiffness has the most significant impact on the outputs, which is reasonable since stiffness directly relates the magnitude of displacement to stresses and forces. Regarding the outputs, they can be categorized as wound area, specific time point and collagen density ratio, which is defined as follows:

$$\begin{aligned} {\hat{\rho }}_c(t)=\frac{\int _{\Omega _w(t)}\rho _c d\Omega }{\int _{\Omega _w(t)}\rho _c^0 d\Omega }, \end{aligned}$$

where $\rho _c$ is the density of the collagen and $\rho _c^0$ is the density of collagen in the undamaged skin. This indicator illustrates the difference between the injured, which is gradually recovering, and uninjured skin.

In Table 4, an estimate of the Monte Carlo error, defined by the standard deviation of the mean, is shown. As the number of samples increases, one can approximate the relative Monte Carlo error by

$$\begin{aligned} r.e. = \frac{{\hat{\sigma }}}{|{\hat{\mu }}|\sqrt{n}}, \end{aligned}$$

where ${\hat{\sigma }}$ and ${\hat{\mu }}$ are the sample standard deviation and sample mean, respectively, and *n* is the sample size. By the Central Limit Theorem (Klenke 2008), one can obtain immediately that the quantity $\displaystyle \frac{{\hat{\mu }}-\mu }{\sigma /\sqrt{n}}$ follows the standard normal distribution, where $\mu$ and $\sigma ^2$ are the population mean and variance, respectively. Given $\theta , \delta >0$, we suppose

$$\begin{aligned} {\mathbb {P}}\left( \frac{|{\hat{\mu }}-\mu |}{\sigma /\sqrt{n}}\geqslant \theta \right) =\delta , \end{aligned}$$

then

$$\begin{aligned} {\mathbb {P}}\left( \frac{{\hat{\mu }}-\mu }{\sigma /\sqrt{n}}\leqslant \theta \right) =1-\frac{\delta }{2}. \end{aligned}$$

We note that $\displaystyle (n-1) \frac{{\hat{\sigma }}^2}{\sigma ^2}$ follows a $\chi ^2$ distribution with $n-1$ degrees of freedom, and therewith $\displaystyle \sqrt{n}\frac{{\hat{\mu }} - \mu }{{\hat{\sigma }}}$ follows a t-distribution with $n-1$ degrees of freedom. However, as $n\rightarrow \infty$, ${\hat{\sigma }}$ converges to $\sigma$ (consistent and unbiased estimator). Hence, for *n* very large, it follows that

$$\begin{aligned} |{\hat{\mu }} - \mu | \leqslant \frac{{\hat{\sigma }}}{\sqrt{n}} \Phi ^{-1}\left( 1-\frac{\delta }{2}\right) , \end{aligned}$$

where $\Phi (z)$ is the cumulative probability function of standard normal distribution. We write the above inequality in terms of the relative Monte Carlo error (denoted by *r*.*e*.), then one obtains

$$\begin{aligned} \frac{|{\hat{\mu }} - \mu |}{|{\hat{\mu }}|} \leqslant \frac{{\hat{\sigma }}}{\sqrt{n} |{\hat{\mu }}|} \Phi ^{-1}\left( 1-\frac{\delta }{2}\right) = r.e. \cdot \Phi ^{-1}(1-\frac{\delta }{2}). \end{aligned}$$

The above inequality in terms of an interval of significance says that the sample mean resides within this interval with estimated probability of $(1-\delta )$. From Table 4, all the relative Monte Carlo errors are less than 0.006, which indicates that regarding the sample mean, the sample size is sufficiently large.

Compared with the results of Monte Carlo simulations in Peng and Vermolen (2020) (see Table 5 in Appendix) where the phenomenological model is used, for the equilibrium and minimal scar area, the standard deviations from this manuscriptis are both one order of magnitude larger. This shows that the morphoelastic model is more sensitive with respect to the changes of input parameter values.

Most of the correlations obtained in this manuscript for morphoelasticity are consistent with the correlations that were obtained in Peng and Vermolen (2020). The current correlations seem to be a bit more significant, which indicates that the model based on morphoelasticity is more sensitive to parameter variation than the phenomenological model from Peng and Vermolen (2020), whereas the standard deviations of most responses are comparable. In other words, the use of morphoelasticity does not significantly reduce the uncertainty in the model. At least significant ($p < 0.001$) correlations are observed between many of the output and input parameters. Therefore, the scar area after a short amount of time after wounding can be used as indicative for the behavior of the scar area at later times, such as the maximum contraction and the final contraction.

**Fig. 10:**
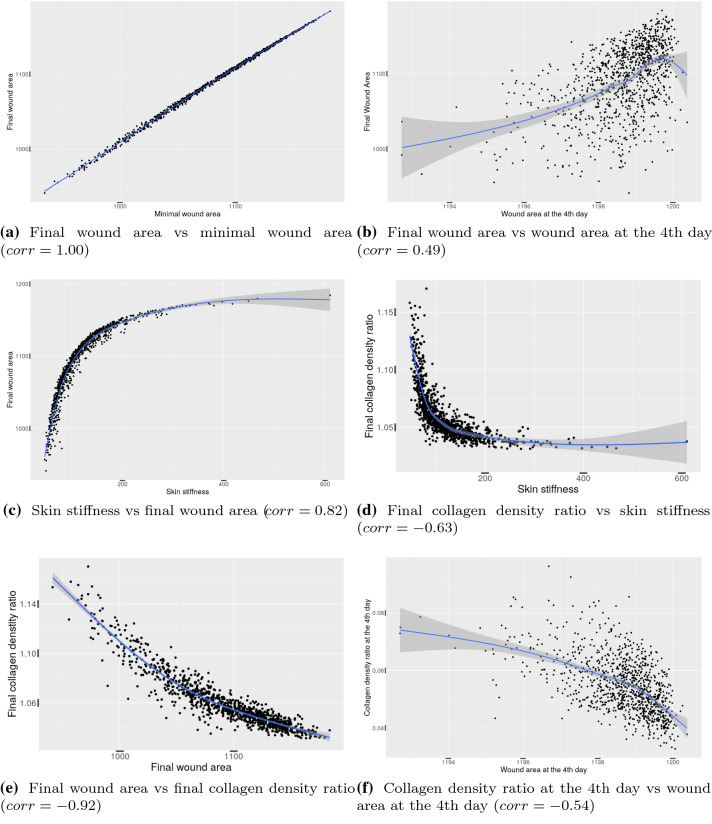
Scatter diagrams of several pairs of variables from the Monte Carlo simulations. Each shaded area represents the $95\%$ confidence interval. In each subplot, the *p* value of the correlation is less than 0.001

Figure 10 shows some scatter plots between various parameters. From Fig. 10a, it can be seen that the correlation coefficient is (almost) equal to one, which reflects a very strong correlation between the maximal contraction and the final contraction. In other words, the more the scar contracts, the more the long-term area differs from the initial area. In other words, there is less absolute recovery in the long term if the earliest maximum contraction is more severe. Furthermore, there is a slightly stronger correlation between the final scar area and the scar area on the fourth day post wounding (see Fig. 10b), compared to the results in Peng and Vermolen (2020), which were based on the phenomenological model for morphoelasticity. This slightly stronger correlation may be a consequence from the fact that the morphoelasticity approach directly connects the scar deformation to the strain tensor. These correlations are useful for clinicians to predict the final contraction of the injured skin.

The stiffness of skin significantly determines the extent of contraction. It has been found that a wound in the buttock contracts more than a wound in the scalp region (O’Leary et al. 2008). In Fig. 10c, it can be seen that the model reproduces this biological observation, for example, it is observed in O’Leary et al. (2008) that the scar contracts less in the scalp (the stiffness is in the order of $20 - 40MPa$ (Trotta and Annaidh 2019)) than in the buttock (the stiffness is in the order of 1*MPa* (Wang and Lakes 2002)). Subsequently, more wound contraction goes with higher collagen density ratio. Therefore, there is a negative correlation between the collagen density ratio and the skin stiffness; see Fig. 10d.

Another difference of the results from the phenomenological model in Peng and Vermolen (2020) is the correlation between the final wound area and the final ratio of average collagen density. In Peng and Vermolen (2020), even though the correlation is significant (i.e. p value is less than 0.001), the scatter plot is not monotonic, therefore, the negative correlation is not convincing to some extent. However, in the current morphoelasticity study, we observe, among other correlations, a significant correlation between the scar area and the ratio of density of collagen. In Fig. 10e, the final wound area and the final ratio of average collagen density are strongly negatively correlated. In other words, higher collagen density results in a higher degree of contractions (i.e. a smaller final scar area). This also holds for the data from the 4th day post wounding; see Fig. 10f. This dependence is caused by the fact that the orientation of the collagen bundles guides the migration direction of the (myo)fibroblasts, and since the (myo)fibroblasts are responsible for the regeneration of collagen bundles, as well as for the contractile forces within the scar, the combination of proliferation of (myo)fibroblasts and remodeling of collagen lead to more contraction. This is also observed in Thomas (2001).

**Fig. 11:**
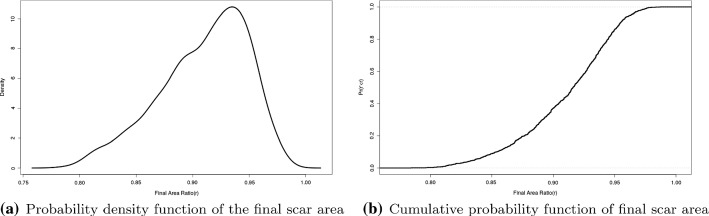
Probability density function and cumulative probability function of the final scar area

To determine the degree of contraction of post-burned skin, the scar area is used as a quantifier. Figure 11 shows the estimated probability density distribution of the final wound area from the Monte Carlo simulations. It is obvious from the shape of this probability density distribution that the final scar area is not normally distributed. A normality test confirms this observation quantitatively. From Fig. 11b, it can be concluded, that considering a wound with size $20\mu m\times 15\mu m$, the probability that the wound will develop $10\%$ contraction is around $40\%$ and $15\%$ contraction is around $10\%$.

## Conclusions

In this manuscript, we consider a formalism to describe the permanent deformation of a scar region by combining morphoelasticity and a cell-based wound healing model. We confirm that both the phenomenological approach and the morphoelasticity approach qualitatively give similar numerical results. Hence, both model formulations can be used to describe to local plasticity of the wound. The parameter sensitivity analysis shows that the viscosity part in the viscoelastic model for the displacement has a significant damping effect on the evolution of the scar area.

Compared to the phenomenological approach, the model with morphoelasticity is more sensitive to the random processes and parameter variations. Furthermore, with the same magnitude of the cellular forces, the model with morphoelasticity predicts larger contractions. Regarding computation time, the solution of the partial differential equations is more expensive in the case of morphoelasticity than for the phenomenological model. However, for large numbers of cells, this difference decreases, which implies that the computation time becomes predominantly determined by the cellular processes. These cellular processes entail the cellular force interaction, development of cellular forces, cell proliferation and cell migration. Based on these considerations, the incorporation of morphoelasticity into the modeling framework is also promising in real-world situations where a parallel computing environment is used.

Next to this deterministic parameter variation, a Bayesian (Monte Carlo) approach has been done for the morphoelastic model. Most of the results confirm the correlations that were obtained from the Monte Carlo simulations on the basis of the phenomenological model. However, the correlation between the ratio of average density of collagen and the scar area is more negatively pronounced in the morphoelastic model. This observation is consistent with the clinical observations in O’Rourke et al. (2015) and in Thomas (2001). For this reason and because of the physics-based nature of morphoelasticity, the model based on morphoelasticity is preferred for use in future research.

Regarding further research, the focus should be directed towards application of the model to an enlargement of the scale and extension to three spatial dimensions, so that the modeling framework is applicable to clinical situations. This enlargement of the modeling scale requires a parallel computing environment. Furthermore, upscaling of the agent-based model to a fully continuum-scale computational framework is also a sensible step.

## Supplementary Information

Below is the link to the electronic supplementary material.

Supplementary file1 (DOCX 5 kb)

Supplementary file2 (MP4 17571 kb)

## Appendix: Results of Monte Carlo simulations with the phenomenological model

To compare with the results of Monte Carlo simulations with the morphoelastic model shown in Sect. 6 (see Table 4), we exhibit the results from Peng and Vermolen (2020) here:

**Table 5:** Distributions of the input parameters in Monte Carlo simulations with the phenomenological model (Peng and Vermolen 2020)

Input Parameters			
Parameter	Description	Distributions	
$E\_s$	Substrate elasticity	log normal$(\log 50, 0.1)$	
$\lambda _d$	Division rate of regular fibroblasts related to strain energy density	uniform(1.5, 2.5)	
$\lambda _a$	Apoptosis rate of myofibroblasts	normal(10, 0.1)	
$\lambda _{immune\_random}$	Rate of Point Poisson Process of macrophages random appearing on the edge between the wound and undamaged skin	log normal$(\log 0.04,10^{-5})$	

Responses/Outputs			
Parameter	Description	Mean (Standard Deviation)	Monte Carlo Error
*n*	The time when the model reaches equilibrium	626.4(38.46908)	1.10591
$n\_min$	The time when the wound has minimal volume	380.1(27.64454)	0.79472
$Area\_final$	The equilibrium wound area	1142(4.658205)	0.13391
$Area\_min$	The minimal wound area	1090(8.324712)	0.23932
$Area\_4days$	The wound area at 4th day after wounding	1198(1.448219)	0.041633
$rho\_c\_hat\_final$	The ratio of average density of collagen when the model reaches equilibrium	0.9950(0.003062998)	$8.8055^{-5}$
$rho\_c\_hat\_min$	The ratio of average density of collagen when the wound area is minimal	0.9336(0.02632869)	$7.56897\times 10^{-4}$
