# Supplementary material for: Comparison between a phenomenological approach and a morphoelasticity approach regarding the displacement of extracellular matrix
Source: Biomech Model Mechanobiol. 2022 Apr 10;21(3):919–35. doi: 10.1007/s10237-022-01568-3 (PMC9132877; doi:10.1007/s10237-022-01568-3)
Supplement: Supplementary file 1 — Supplementary file1 (DOCX 5 kb) [file 10237_2022_1568_MOESM1_ESM.docx]

**Supplementary material**

Video 1:

In the video, the deformation of the scar region (the subdomain in the middle of the computational domain) has been shown. The black curve represents the original scar region and the red curve represents the deformed scar. Different colors of dots are different cell phenotype: red dots are macrophages (immune cells), blue dots are fibroblasts and black dots are myofibroblasts.
